# Supplementary material for: Genetic comparisons of interleukin-17 reveal a framework for complex signaling evolution
Source: bioRxiv. 2026 Apr 14:2026.04.13.718218. Preprint. [Version 1] doi: 10.64898/2026.04.13.718218 (PMC13105070; doi:10.64898/2026.04.13.718218)
Supplement: Supplement 3 [file media-3.zip › Supplemental files for paper/ERC/IL17xIL17_comparisons/IL17REL_IL17RD.pdf]

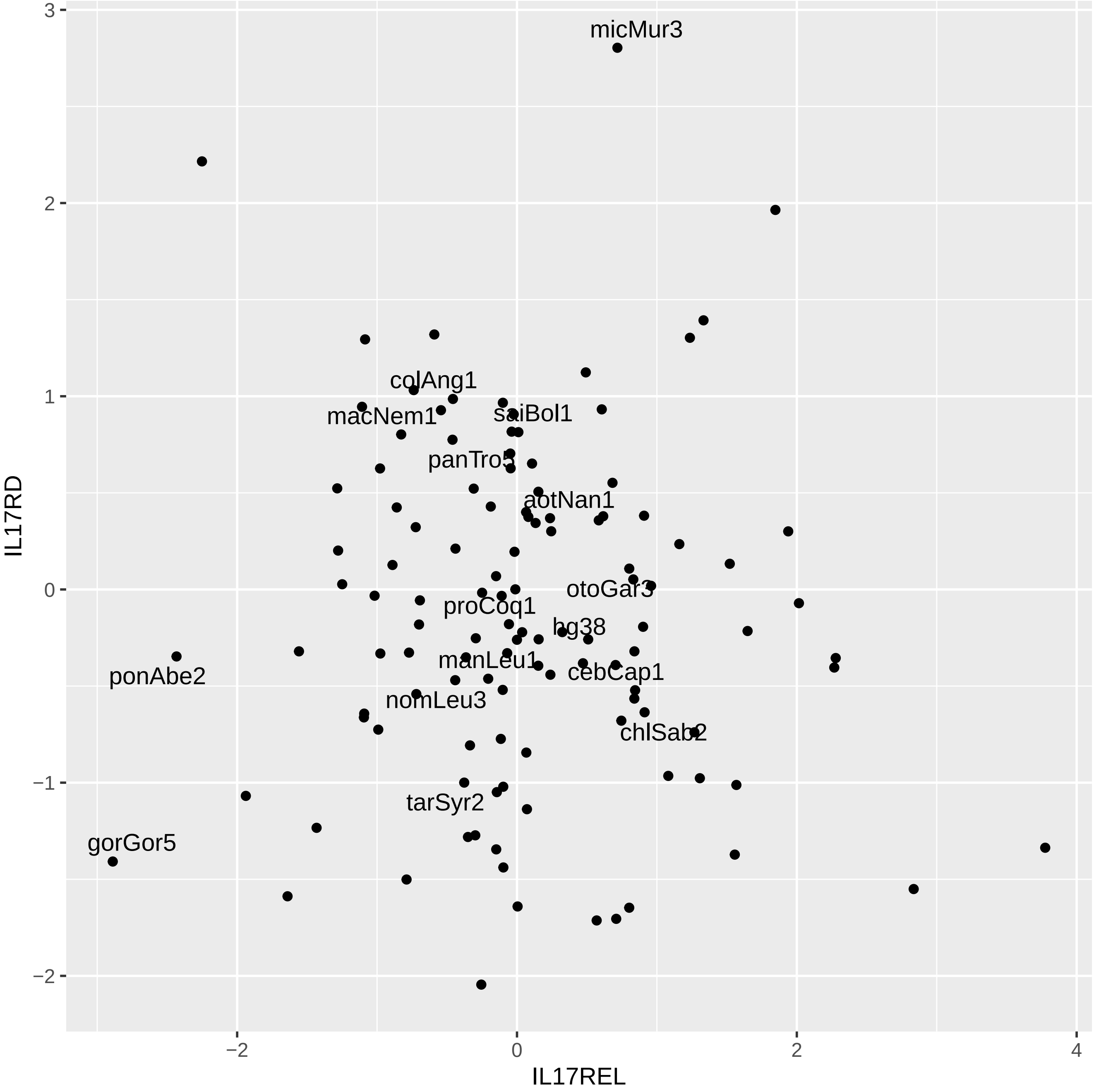

micMur3
colAng1
macNem1
saiBol1
panTro5
aotNan1
otoGar3
proCoq1
hg38
manLeu1
cebCap1
ponAbe2
nomLeu3
chlSab2
tarSyr2
gorGor5
3
2
1
0
−1
−2
−2
0
2
4
IL17RD
IL17REL
